# How to remain in working life with hearing loss – health factors for a sustainable work situation

**DOI:** 10.3233/WOR-230377

**Published:** 2024-11-08

**Authors:** Sarah Granberg, Stephen Widén, Johanna Gustafsson

**Affiliations:** a School of Health Sciences, Örebro University, Örebro, Sweden; b Faculty of Medicine and Health, Örebro University, Örebro, Sweden

**Keywords:** Hearing loss, working conditions, workplace, health, occupational health, salutogenesis

## Abstract

**BACKGROUND:**

Persons with hearing loss (HL) are a vulnerable group in working life. Studies have shown that they are more likely than the general population to be in part-time work, to be unemployed, receive disability pension, and to be on sick leave. Many workers with HL also experience unhealthy work conditions, such as jobs where they experience high demands combined with low control as well as safety concerns and social isolation. There is a lack of studies that focus on factors that promote a healthy, sustainable work situation for the target group.

**OBJECTIVE:**

To investigate health factors that contribute to a sustainable work situation for employees with HL.

**METHODS:**

The current study was a comparative, observational study with a cross-sectional design including a clinical population of adults with HL. Comparisons were made between workers with HL “in work” and workers with HL on “HL-related sick leave”.

**RESULTS:**

Seven health factors were identified. Those “in work” experienced a healthier work environment as well as lower levels of mental strain, hearing-related work characteristics and content, cognitively demanding work content, hearing-related symptoms, energy-demanding activities, and bodily aches and pain than those on “HL-related sick leave”.

**CONCLUSION:**

The results demonstrate a clear pattern regarding health factors for a sustainable working life. The type of job was not related to whether an individual was on sick leave or working. Rather, the work climate and the content of the work mattered.

## 1 Introduction

Healthy working life, where the health, safety and well-being of workers are promoted, are globally recognized as a prioritized area [1] for all workers (i.e., Article 23 of The Universal Declaration of Human Rights [2]), including workers with disabilities (i.e., article 27 of The Convention of Rights for People with Disabilities [3]).

### 1.1 Hearing loss in working life

A healthy working life can be a challenge for persons with hearing loss (HL). Compared to others in the general population, persons with HL may find themselves in a precarious position in working life because they are more likely, as a group, to be in part-time work [4], to receive disability pension [5], to be unemployed [6], and on sick leave [7]. Employees with HL are also more likely to experience unhealthy work conditions, such as jobs where they experience high demands in combination with low control [8] as well as safety concerns and social isolation [9]. Furthermore, qualitative studies exploring the job-related experiences of employees with HL indicate that HL affects work ability due to difficulties in interaction and communication [10]. Other job-related experiences involve emotional well-being and energy levels; studies have revealed that HL has an impact on employees’ sense of exclusion, withdrawal, and fatigue [11], lack of energy [12] and job-related exhaustion [13]. However, the evidence about job-related well-being and energy levels among employees with HL is somewhat unclear; other studies have reported that employees with HL do not show reduced emotional well-being or a higher need for recovery after work [14]. A mediating factor might be work characteristics. Nachtegaal et al. [15] found a relationship between psychosocial work characteristics and the need for recovery, where hearing status was a nonconfounding factor. The need for recovery covaried with job demands and job control: the higher the job demands and the lower the job control, the greater the need for recovery. This finding is in line with Danermark et al. [8], who concluded that in most aspects, the work experiences of employees with HL followed the same pattern as for the general population, although the magnitude of problems was larger for employees with HL. The research points to individual experiences of functioning, well-being, and health in the working life of employees with HL and describes an interaction between individual factors, such as HL, and work situations that provide health-promoting job characteristics. However, the research in this field is inconclusive. Some studies find that people with HL are at greater risk of experiencing problems related to work life than the general population, while other studies suggest that this is not always the case. Hence, there is a need for more research to determine the specifics of what promotes or hinders work-life well-being for people with HL. It is clear that a substantial part of research on HL and working life has focused solely on the negative consequences of HL in relation to employment and work and hearing-related challenges for employees with HL [16]. Consequently, the amount of research that investigate factors that promote a healthy working life for persons with HL is sparse.

### 1.2 Healthy working life

Since the beginning of the 21st century, theoretical discussions regarding good health and ill health have been dominated by suggestions that ill health and good health are nondichotomous concepts. This means that if one “removes” factors of ill health from an individual, this does not necessarily result in improved health. Consequently, good health goes beyond the absence of ill health. Studies that have applied this fundamental assumption to health in working life [e.g., 17–19] have demonstrated a complex pattern with regard to understanding a healthy working life in which aspects at work matter in addition to individual factors and aspects of family life (i.e., life outside work). In sum, studies demonstrate that the work environment is an important health factor, and support from managers and job designs that facilitate energy balance and thus provide resources “to do a good job” are important. Furthermore, opportunities for recovery both at work and at home also matter [18]. Consequently, a healthy working life must be viewed from a multidimensional perspective where the entire life situation of an individual is considered. Aronsson [19] concluded that aspects related to health psychology would deepen knowledge of the factors that constitute health in the workplace. As a direct result of Aronsson’s study, a questionnaire that focuses on health in the workplace was developed, the Work Experience Measurement Scale (WEMS) [20, 21]. Based on several health psychology theories, such as the job demand-control-support (JDCS) model [22] and the effort-reward-imbalance (ERI) model [23], the WEMS presents a multidimensional view of the work experience.

Several studies have highlighted the correlation between personality traits and individual experiences of health. Gustavsson [24] constructed a short-form inventory of health-relevant personality traits and health, the hp5 inventory (hp5i). Based on the five-factor model (FFM), the inventory is applicable in health research. This inventory contains five dimensions: antagonism, impulsivity, hedonic capacity, negative affectivity, and alexithymia. All the dimensions are defined as health-relevant facets of the original personality constructs of the FFM. Several of these dimensions are correlated with health. Hedonic capacity in particular has a clear relation to good health. Positive affectivity has been identified as a core dimension of a hedonic capacity (i.e., the original FFM construct extraversion) and represents a positive mental approach toward daily activities [25]. Gustavsson et al. [24] state that hedonic capacity may explain why some individuals react and adapt in a more engaged and less anxious way than others when exposed to stressors in daily life. Regarding working life, several studies have focused on behaviors in relation to attendance at work and have established an association between the FFM construct of neuroticism (such as negative affectivity [24]) and sick leave, where neuroticism was associated with more occasions of sick leave and/or number of sick leave days [e.g. 26, 27]. However, in a cross-sectional study including 364 participants, Østby et al., [28] found no *statistical* association between personality and long-term sick-leave. However, as the authors noted, high neuroticism and low extraversion was associated with long-term sick leave.

#### 1.2.1 Salutogenesis

An important concept in relation to health is salutogenesis. Salutogenesis was originally introduced by Aaron Antonovsky and refers to life experiences that support the mobilization of coping resources [29]. A salutogenic orientation thus focuses on positive outcomes and their underlying resources rather than focusing on negative outcomes and their underlying risk factors [30]. In research on work, the salutogenic approach focuses on health-related outcomes and “strives to understand the underlying mechanism of (positive) health development at work” [31, p. 332]. Studies from a salutogenic perspective on working conditions for employees with HL have primarily examined strategies to *manage* the work situation [e.g., 10–12, 32–34]. These studies point to a range of salutary factors, including support from colleagues and management, availability of workplace accommodations, effective communication strategies, disclosure of HL, individual coping abilities and self-accommodation, that are commonly described as facilitators of a manageable work situation. There is, however, a clear lack of studies that focus on a healthy WL from a multidimensional perspective, i.e., where the complexity of HL, health factors and working life aspects are captured.

### 1.3 Objectives

Given that the work experiences of people with HL demonstrate complex interactions between individual and organizational factors [16], and that a multidimensional, salutary perspective is essential when studying health at work [30], the aim of the current study was to investigate health factors that contribute to a sustainable work situation for employees with HL. Two research questions were addressed in this study:

1. What kind of HL-related personal and work life factors can be considered “health factors” for employees with HL?
2. Can these factors be associated with differences in position on the labor market (i.e., in work or on HL-related sick leave)?

## 2 Methods

### 2.1 Study design

The current study was a comparative observational study with a cross-sectional design that included a clinical population of adults with HL.

### 2.2 Population

All 2930 adults of working age registered at the audiological clinic in Örebro County, Sweden, in 2018 were invited to participate in the current study. Study participation involved answering a survey about HL in working life. The inclusion criteria were as follows: 18–67 years of age, registered patient at the audiological clinic (i.e., established HL), currently working in paid work (part time or full time) or currently (or during the last year) on sick leave from paid work. A total of 495 persons responded to the survey. The internal drop-out rate varied between questions in the questionnaire.

The age span in the current study was determined based on the general working age in Sweden (18–67 years). However, the official retirement age is a statutory regulation; in reality, it is common for people to retire from work earlier than the age of 67. The audiological clinic does not maintain registers of its patients’ positions on the labor market, so whether a person works cannot be determined beforehand. Consequently, it is likely that a large number of the invited participants (*n* = 2930) did not fulfill the inclusion criteria regarding employment status; hence, the size of the background population is impossible toidentify.

### 2.3 Materials

**Fig. 1:**
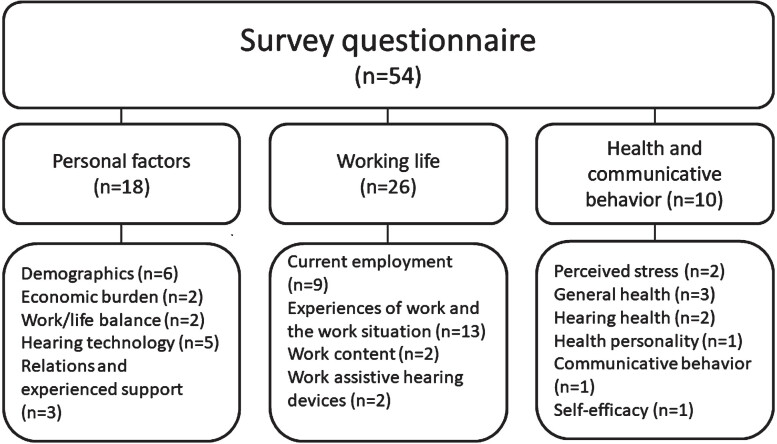
Overview of the survey questionnaire, n=number of variables.

The survey contained a developed questionnaire of 54 variables. These variables were single questions, overarching questions containing sub questions (such as statements), or established indices. A thorough literature review on health in working life and health/ill health in relation to HL determined the focus of the questionnaire. Based on this literature review, three subareas (personal factors, work life, health and communicative behavior) were addressed in the questionnaire (Fig. 1).

The vast majority of the questions/indices were used in other research studies, in public labor market surveys by Statistics Sweden, or in public health studies by the Public Health Agency of Sweden. Permission to use these questions/indices was obtained from the different agencies, authorities, and/or authors.

#### 2.3.1 Personal factors

This subarea contained 18 variables. The questions covered *demographic aspects* such as gender, age, educational level, and living conditions. *Economic burden* was assessed with two questions. This concept addresses the economic resources of an individual to handle unforeseen costs. Financial situation has been found to have a connection to health and well-being [35]. The questions have been used in numerous population studies in Sweden [36, 37]. *Work-life balance* was assessed with two questions that focused on recovery in everyday life based on findings by Nachtegaal et al. [15] and on the domestic burden (the burden outside work that consumes energy) based on Aronsson [19]. Five questions were asked regarding *hearing technology*, including the number of hearing aids (HA)/cochlear implants (CI) an individual owned, the extent to which the individual used his or her HA, and the experienced positive impact on quality of life with HA/CI. *Relations and experienced support* were assessed with three questions that focused on the quality of the relations with close family and friends, support from family and friends and whether individuals considered themselves to have a close friend [36].

#### 2.3.2 Work life

This subarea contained 26 variables. The questions regarding *current employment* concerned the type of employment (e.g., employee, self-employment), sick leave, type of job, and the time the individual currently worked (percent of full time). Because “type of job” was a free-text question, the answers were later coded in relation to the Swedish Standard Classification of Occupations, the SSYK [38]. Classifications were made in relation to the ten overarching sectors in the SSYK. Because most of the respondents in the HL-related sick leave group were employed in sectors two and five (2. Occupations requiring an advanced level of higher education and 5. Service, care, and shop sales workers), these two sectors were further classified according to the following subsectors: Medical care (e.g., nurses and medical doctors), Education (e.g., preschool teachers, teachers), Social work (e.g., social workers, counselors), Sales (e.g., shop assistants, sales clerks) and Health and social care (e.g., caregivers). *Experiences of work and the work situation* included the Work Experience Measurement Scale (WEMS) [20, 21]. The WEMS contains 32 statements divided into six dimensions of work experience: supportive working conditions, internal work experience, autonomy, time experience, management, and reorganization. Additional index questions were retrieved from the Labor Force Survey (LFS) focusing on leadership, competence development, and wage developments [37]. *Work content* focused on work aspects known to be difficult from an HL perspective. This subdimension contained questions based on working-life studies by Kramer et al. [39], Danermark et al. [8], and the LFS [37]. The respondents had to decide whether their work situations consisted of, for example, large amounts of oral communication, conversations with unfamiliar persons/voices, demanding problem solving, and tasks in relation to sound localization and sound discrimination. A few questions related to *work-assistive hearing devices* and whether individuals used any kind of assistive hearing device in their working life in addition to HA/CI (e.g., telephone amplifier, FM systems for group conversations).

#### 2.3.3 Health and communicative behavior

This subarea contained 10 variables. *Perceived stress* focused on stress in relation to the whole life situation and contained questions retrieved from the annual Swedish population survey “Health on Equal Terms” [36]. *General health* was assessed by a subjective estimation of general health and assessments of health status. Health is somewhat difficult to measure because of the ambiguous interpretation of the concept, i.e., what constitutes health is a highly subjective matter. Because working life may negatively impact health (i.e., psychological and physical ill health and/or pain), different health aspects were valued as important in the current study. The questions were based on the LFS [37] and the “Health on Equal Terms” survey [36]. Questions regarding *hearing health* concerned health issues that are known to be comorbid with HL, such as tinnitus and vertigo. Fredriksson et al. [40] found that the concept of “sound-induced auditory fatigue” constitutes a major health problem in working life. This question was considered important to incorporate into the current survey questionnaire. In addition, hearing health questions were retrieved from the Public Health Agency [36]. *Communicative behavior* was assessed by the Communication Strategy Scale [41], a person-report outcome measure that assesses the use of communication strategies. The scale contains 24 items distributed in three subdomains: verbal strategies, nonverbal strategies, and maladaptive behaviors. The scale was used as specified by the scale instructions, i.e., items from each subdomain were merged into indices. Because of the known relationship between health-relevant personality traits and health (see introduction section), the hp5i [24] was used to assess *health personality.* The inventory contains 20 items divided into five subdomains: antagonism, impulsivity, hedonic capacity, negative affectivity, and alexithymia. The inventory was used according to the instructions, and the 20 items were merged into five indices.

### 2.4 Procedure

Initially, the survey was meant to be an internet survey. An invitation letter, including a letter of consent and a web link to the survey, were sent to the 2930 potential participants. Due to the low response rate, a reminder in paper format was sent to nonresponding individuals. All respondents who chose to participate returned signed consent. Persons who responded to the survey but did not return the consent were removed from the study after two reminders.

The sample was divided into two groups for comparison. The first group consisted of persons currently working in paid work (part time or full time), and the other group comprised persons currently (or during the last year) on HL-related sick leave (part time or full time). It is difficult to obtain official statistics regarding HL-related sick leave in Sweden because many persons with HL receive certificates of work absence in relation to another, or a comorbid, health condition such as severe stress, fatigue syndrome or depression. This issue is likely a consequence of the fact that many persons with HL receive their sick notes from their GP and not from an audiological/ENT physician. Consequently, in the current survey, a specific question was asked to participants who claimed to be on sick leave regarding whether they considered the specified sick leave to be associated with HL-related issues. Only participants who answered this particular question affirmatively were included in the sick-leave group. The HL-related sick leave group included 60 participants.

### 2.5 Analyses

Initially, exploratory factor analysis (EFA) of variables was performed to detect interrelationships and to identify independent latent variables. Principal component analysis (PCA) was chosen as the factor extraction method, and varimax with Kaiser normalization was chosen as the rotation method. Based on Hair et al. [42] and the scree plot of the EFA, 12 principal components were retained that explained 60.6% of the variance. According to Hair et al., loadings should be 0.3 and higher. Consequently, loadings under 0.3 were removed from the analysis (Table 1). Although the variables in each factor belonged to the same construct, they measured and focused on different aspects. One such example from factor one is the variables “diagnosed depression” and “self-efficacy”, which are closely related to psychological aspects and therefore load in the same factor; however, they measure quite different aspects. Consequently, to avoid loss of data information, no indices were created based on all variables in the same factor. Cronbach’s alpha (*α*) was calculated for each factor and had to reach a value of 0.6 to remain in the factor analysis.

**Table 1:** Principal component analysis and an eight-factor solution (varimax rotation, n = 495)

F1	F2	F3	F4	F5	F6	F7	F8	Com.
Anxiety &worry	.735								.717
Thoughts &emotions (index)	.734								.790
Negative affectivity (index, HP5I)	.674								.679
Self-efficacy	.660								.602
Perceived stress	.610								.608
Difficulties concentration	.590								.590
Depression (diagnosed)	.563								.665
Hedonic capacity (index, HP5I)	-.558								.666
Relations with family &friends	.548								.581
Rest &recovery in everyday life	.484								.634
Balance between work &spare time	.470								.550
Difficulties sleeping	.469								.562
Irritation &anger	.364								.567
Supportive working conditions (index, WEMS)		.825							.826
Management (index, WEMS)		.823							.726
Competence development (index)		.820							.806
Reorganization (index, WEMS)		.790							.709
Internal work experience (index, WEMS)		.732							.793
Satisfaction with salary (index)		.574							.625
Autonomy (index, WEMS)		.552							.536
Sudden changes in sound environment			.864						.779
Distracting sounds affecting concentration			.824						.753
Loud noise levels at work			.785						.749
Sound localization			.745						.706
Bothered by noise at work			.702						.711
Sound discrimination			.687						.689
Pay attention to sounds/alarms			.586						.658
Demanding problem solving				.744					.706
High level of concentration				.731					.649
Conversations with unfamiliar people &voices				.564					.595
Pressure of time (index, WEMS)				-.544					.682
High degree of spoken communication				.440					.527
Tinnitus					.689				.592
Sound fatigue					.651				.688
Headache or migraine					.527				.544
Fatigue					.453				.573
Dizziness					.444				.476
Non-verbal communication strategies						.820			.743
Verbal communication strategies						.816			.792
Energy demanding tasks outside work						.395			.436
Backpain							.767		.640
Pain in shoulders or neck							.571		.595
Pain in elbows, legs, or knee							.533		.603
Someone to share feelings and thoughts with								.775	.689
Someone to get help from if needed								.587	.606
KMO=.817
Bartlett’s Test = 5359.41^***^
Cronbach's Alpha	0.867	0.868	0.896	0.718	0.724	0.618	0.649	0.374
Eigenvalue	10.60	5.13	2.61	2.44	2.33	1.93	1.85	1.62	28.51
Variance %	19.99	9.68	4.92	4.61	4.39	3.64	3.48	3.05	53.76

The variables in each factor were compared in relation to the nondependent groups “HL-related sick leave” and “in work”. The nonparametric Mann–Whitney U test was chosen for the analysis of group differences. Effect size (ES) was calculated for each comparison by using the formula suggested by Rosenthal $\left(r=\frac{z}{\sqrt{N}}\right)$ that is suitable for nonparametric tests with two independent samples. The *r* correlation coefficient was interpreted using Cohen's rules of thumb: 0.1, small effect; 0.3, medium effect; and 0.5, large effect.

Patient characteristics were analyzed with frequency analysis including the chi-square test to establish group differences in relation to expected values.

## 3 Results

### 3.1 The sample

Table 2 describes the demographic variables, HA and related information, and accommodations at work.

**Table 2:** Demographics and hearing technology characteristics

Compared variables including level of significance		Frequency (absolute, relative)			
		In work		HL-related	
				sick leave	
Sex^*^ (*n* = 468)
	Men	200	48.9 %	21	35.6 %
	Women	209	51.1 %	38	64.4 %
Age (years)^*^ (*n* = 469)
	18-29	26	6.4 %	6	10.0 %
	30-54	134	32.8 %	29	48.3 %
	55-67	249	60.9 %	25	41.7 %
**Level of education** *ns* (*n* = 468)
	Elementary and lower secondary school	42	10.3 %	3	5.0 %
	Vocational secondary school	93	22.8 %	8	13.3 %
	Upper secondary school	86	21.1 %	14	23.3 %
	Folk high school	9	2.2 %	3	5.0 %
	University education < 3 years	60	14.7 %	10	16.7 %
	University education≥3 years	118	28.9 %	22	36.7 %
**Economic burden** *ns* (*n* = 465)
	Difficulties handling running costs (yes)	25	6.2 %	6	10.2 %
	Difficulty dealing with an unexpected expense (yes)	42	10.4 %	11	18.6 %
**Type of job; sectors 2, 5, other** *ns* (*n* = 469)
	Medical care	32	7.8%	4	6.7%
	Education	41	10.0%	6	10.0%
	Social work	6	1.5 %	3	5.0%
	Sales	15	3.7 %	2	3.3 %
	Health and social care	47	11.5 %	10	16.7%
	Other (not sectors 2 &5)	268	65.5 %	35	58.3 %
**Employment rate** ^*^ (*n* = 469)
	Full time (100 %)	262	64.1 %	32	53.3 %
	Part time (75 %)	28	6.8 %	10	16.7%
	Other	119	29.1 %	18	30.0 %
**Hearing Aids (HA)** *ns* (*n* = 465)
	Yes	299	73.6 %	44	74.6 %
	No	107	26.4 %	15	25.4 %
Cochlear Implant/s (CI)^***^ (*n* = 465)
	Yes	12	3.0 %	9	15.3 %
	No	394	97.0 %	50	84.7 %
**Usage of HA/CI** *ns* (*n* = 345)
	Daily basis	234	78.0%	36	80.0%
	Occasionally	45	15.0 %	6	13.3 %
	Never	21	7.0%	3	6.7 %
**HA/CI positive impact on QoL** *ns* (*n* = 351)
	Yes	247	81.0 %	33	71.7 %
	Sometimes	48	15.7 %	10	21.7 %
	No	10	3.3 %	3	6.5 %
**Assistive hearing devices at work** *ns* (*n* = 465)
	Yes	103	25.4 %	22	36.7 %
	No	302	74.6 %	38	63.3 %

^*^ = *p* <  .05 ^**^=*p* <  .01 ^***^ = *p* <  .001 ns = nonsignificant.

A total of 468 respondents answered the question regarding sex affiliation, and there was an even distribution regarding sex for the entire sample. However, when investigating the sex distribution in relation to the two groups, significantly more women than men were found in the HL-related sick leave group (*p* <  .05).

Regarding age, the majority of respondents in the “HL-related sick leave” group were younger, i.e., 18-54 years of age (58.3%), compared to the “in work” group, where 60.9% of the respondents were 55-67 years of age (*p* <  .05). All respondents in the sample were highly educated, and most respondents (regardless of group affiliation) had a university education longer than 3 years as their highest level of education.

Most respondents worked in the “health and social care” sector. No significant differences regarding the type of job were detected between the two groups. There were significant differences between the two groups regarding the employment rate (*p* <  .05). Although most respondents worked full time, more respondents in the “HL-related sick leave” group worked part time (i.e., 75% of full time) compared to those “in work”.

The two groups were similar regarding ownership of HAs (monaural or binaural): 73.6% (in work) and 74.6% (HL-related sick leave). With regard to how often the respondents used their HAs, 80.0% in the HL-related sick leave group used their HAs on a daily basis compared to 78.0% in the in-work group. No statistically significant differences were detected between the groups regarding HA use. Only 22 individuals in the entire sample used CIs. Of these respondents, 9 individuals belonged to the group “HL-related sick leave”. The respondents were asked to evaluate whether they thought their HAs or CIs influenced their quality of life (QoL) in a positive way. The majority of the respondents agreed with this statement, but no significant differences were detected between the two groups.

Regarding the use of assistive hearing devices at work, no statistically significant differences were detected between the two groups. Notably, only 26.9% of the entire sample used this type of accommodation at work.

### 3.2 Health factors for a sustainable work situation

Fifty of the variables in the questionnaire were analyzed with factor analysis. The analysis indicated an eight-factor solution (45 variables) with factors with an eigenvalue above 1.0 that explained approximately 54% of the variance (Table 1). The interpretation of the eight factors was “Mental strain” (F1, 13 variables), “Work environment” (F2, 7 variables), “Hearing-related work characteristics and content” (F3, 7 variables), “Cognitively demanding work content” (F4, 5 variables), “Hearing-related symptoms” (F5, 5 variables), “Energy-demanding activities” (F6, 3 variables), “Bodily pain and ache” (F7, 3 variables) and “Social support” (F8, 2 variables). Cronbach's alpha demonstrated acceptable values for Factors F1-F7 but low values for Factor F8, indicating low correlation between the items in F8. Consequently, Factor 8 was removed from further analysis.

Table 3 demonstrates the comparative analyses of Factors 1-7 between the two groups “in work” and “HL-related sick leave”. When analyzing potential differences between the two groups, significant differences were found for all items in the factor “Mental strain” (Factor 1) except for self-efficacy and hedonic capacity. The result reveals a general pattern where individuals in work scored lower on items associated with mental strain, indicating that they experienced fewer problems with, for instance, anxiety, perceived stress, difficulties concentrating, diagnosed depression and the balance between work and spare time. Furthermore, they scored low regarding the health personality trait of negative affectivity. Although not significant, persons “in work” scored high for the variable of hedonic capacity (reversed loading), i.e., a tendency to hold a less anxious approach to experienced stressors in life among those “in work”.

**Table 3:** Comparisons of the two groups “in work” and “HL-related sick leave”

**Factor 1, Mental strain**	Sick leave	mean rank (*n*)	In work	mean rank (*n*)	Z	ES
Anxiety &worry	59	287.42	404	223.91	–3.77^***^	0.18
Thoughts &emotion (index)	59	299.21	401	220.39	–4.27^***^	0.20
Negative affectivity (index)	59	319.74	396	214.33	–5.78^***^	0.27
Hedonic capacity (index)	59	218.55	398	230.55	–0.66 ns	0.03
Self-efficacy (index)	57	252.43	390	219.84	–1.79 ns	0.08
Perceived stress	59	303.70	406	222.73	–4.75^***^	0.22
Difficulties concentrating	59	321.10	404	218.99	–6.03^***^	0.28
Depression (diagnosed)	58	284.54	405	224.48	–5.11^***^	0.24
Relations with family &friends	60	277.04	407	227.65	–3.26^**^	0.15
Rest &recovery in everyday life	59	285.61	408	226.54	–3.77^***^	0.17
Balance between work &spare time	60	290.75	409	226.82	–4.08^***^	0.19
Difficulties sleeping	59	287.79	404	223.85	–3.69^***^	0.17
Irritation &anger	59	292.02	405	223.83	–4.26^***^	0.20
**Factor 2, Work environment**
Supportive working conditions (index, WEMS)	56	173.83	362	215.02	–2.38^*^	0.12
Management (index, WEMS)	50	137.35	297	180.17	–2.79^**^	0.15
Competence development (index)	53	138.33	353	213.28	–4.34^***^	0.22
Reorganization (index, WEMS)	51	136.54	319	193.33	–3.53^***^	0.18
Internal work experience (index, WEMS)	56	167.38	392	232.66	–3.54^***^	0.17
Satisfaction with salary (index)	51	167.40	260	211.89	–2.67^**^	0.13
Autonomy (index, WEMS)	56	163.38	388	231.03	–3.70^***^	0.18
**Factor 3, Hearing-related work characteristics and content**
Sudden changes in sound environment	58	278.73	404	224.72	–3.00^**^	0.14
Distracting sounds affecting concentration	60	289.93	403	223.37	–2.72^***^	0.17
Loud noise level at work	60	279.17	407	227.34	–2.88^**^	0.13
Sound localization	60	289.28	403	223.47	–2.67^***^	0.17
Bothered by noise at work	60	284.75	406	225.93	–3.28^**^	0.15
Sound discrimination	58	267.65	399	223.38	–2.45^*^	0.11
Pay attention to sounds/alarms	59	259.28	403	227.43	–1.83 ns	0.08
**Factor 4, Cognitively demanding work content**
Demanding problem solving	59	264.02	394	221.46	–2.36^*^	0.11
High level of concentration	59	285.70	405	224.75	–3.36^**^	0.16
Conversation with unfamiliar people &voices	60	249.55	405	230.55	–1.05 ns	0.05
Pressure of time (index, WEMS)	60	286.24	405	225.11	–3.30^***^	0.15
High degree of spoken communication	59	259.44	404	227.99	–1.75 ns	0.08
**Factor 5, Hearing related symptoms**
Tinnitus	59	269.56	403	225.93	–2.42^*^	0.11
Sound fatigue	59	320.89	404	219.02	–5.69^***^	0.26
Headache or migraine	59	294.98	406	223.99	–4.39^***^	0.20
Fatigue	59	326.04	404	218.27	–6.16^***^	0.29
Dizziness	58	284.46	403	223.31	–3.89^***^	0.18
**Factor 6, Energy demanding activities**
Nonverbal communication strategies	57	274.45	400	222.52	–2.78^**^	0.16
Verbal communication strategies	55	277.32	391	215.93	–3.31^**^	0.13
Energy demanding tasks outside work	60	265.18	407	229.40	–2.01^*^	0.09
**Factor 7, Bodily pain and ache**
Back pain	58	256.05	406	229.14	–1.51 ns	0.14
Pain in shoulders and neck	59	279.14	404	225.12	–3.04^**^	0.07
Pain in elbows, legs or knee	59	237.91	403	230.56	–0.435 ns	0.02

^*^ = *p* <  .05 ^**^ = *p* <  .01 ^***^ = *p* <  .001 ns = nonsignificant.

Factor two included experiences of the “Work environment”. Significant differences were found for all seven items. The general pattern was that individuals “in work” scored higher on items associated with the work climate, indicating that they experienced more satisfaction with their working climate in comparison to people on HL-related sick leave.

Regarding Factor three, “Hearing-related work characteristics and content”, and Factor four, “Cognitively demanding work content”, the analysis indicated significant differences for all items except “Pay attention to sounds and alarms” for Factor three and “Conversation with unfamiliar voices” and “high degree of spoken communication” for Factor four. The interpretation of the differences between the two groups in Factor three is that adults with HL “in work” experience fewer working life facets where sufficient hearing is beneficial. This interpretation is based on the fact that all the aspects in this factor are issues of working life where the loss of hearing matters, i.e., noise, distracting sounds, sound localization and sound discrimination abilities. In Factor four, individuals “in work” experienced significantly lower cognitively demanding work content compared to those on “HL-related sick leave”. Furthermore, the item concerning “experienced time pressure” also loaded on Factor four. The factor loading was reversed compared to the other items (–), indicating that persons “in work” also experienced less time pressure compared to those on “HL-related sick leave”. Consequently, low cognitive demands in combination with low time pressure seem to be a health factor in the current study. Interestingly, the variable “high degree of spoken communication” demonstrated no significant differences between the two groups. This means that the amount of spoken communication in work is not related to whether one is on “HL-related sick leave”.

Regarding the Factors “Hearing loss-related symptoms” (F5), “Energy demanding activities” (F6), and “Bodily pain and ache” (F7), the results followed the same patterns as the previously presented analyses. The “in work” group reported significantly fewer problems with hearing-related symptoms and engaged significantly less in energy-demanding activities in their lives. In the Factor “bodily pain and ache”, only the item “pain in shoulders and neck” revealed significant differences, with individuals “in work” reporting fewer of these problems.

## 4 Discussion

The current study investigated health factors for a sustainable work situation in relation to HL. When comparing individuals “in work” with those on “HL-related sick leave”, seven health aspects (Factors) were identified. From the results, it was clear that those “in work” experienced less mental strain, a healthier work environment, less hearing-related work characteristics and content, less cognitively demanding work content, fewer HL-related symptoms, less energy-demanding activities and fewer bodily pain and aches compared to those on “HL-related sick leave”. Furthermore, significant differences were found for almost every investigated variable.

Interestingly, the type of job the respondents held did not seem to matter in the current study. No significant differences were identified between the two groups regarding this aspect. Instead, it seems that healthy factors for a sustainable work situation are related to specific workplaces (the working climate) in combination with individual aspects such as hearing and psychological health.

Furthermore, it is noteworthy that so few of the respondents used assistive hearing technology at work. Anecdotal information from clinicians reveals that this type of work accommodation is viewed by many persons with HL as a “lifesaver” and something that enables them to work. In Sweden, assistive hearing devices at work are subsidized by the Swedish Social Insurance Agency (SSIA); hence, the use of this kind of work accommodation is related to SSIAs’ willingness to grant applications for assistive hearing devices at work. Whether the respondents in the current study had applied for grants for assistive hearing devices at work is unknown. It is also unknown whether the clinicians who treated the patients (given that the respondents in the current study were patients at an audiological clinic) highlighted the aspect of assistive hearing devices at work during their clinical encounters. In any case, this aspect might be an essential focus in audiological rehabilitation.

### 4.1 Factor 1, Mental strain

Factor 1 explained approximately 20% of the variance in this study. Furthermore, all included variables in Factor 1, apart from self-efficacy and hedonic capacity, demonstrated significant differences between the two groups at the *p* <  .001 level.

The health personality traits “negative affectivity” (neuroticism) and “hedonic capacity” (extraversion) loaded in this factor. Negative affectivity concerns “the susceptibility to negative emotions and to related behavioral and cognitive characteristics” [24, p. 73], while “hedonic capacity” is the opposite, i.e., a generally positive outlook on life, positive affectivity, and enthusiasm about daily activities [24, 43]. There is an established relationship between personality traits (the five-factor model, FFM) and health, indicating that individuals who score high in neuroticism also score low in subjective and existential well-being and health-related coping behaviors and score high in anxiety aspects [43]. Furthermore, persons who score high in extraversion (hedonic capacity) score high in resilience, health-related coping behavior and social support from peers and family [43]. In the current study, it is clear that people ”in work” scored high in “hedonic capacity” and low in “negative affectivity”. Furthermore, they also scored low in all variables related to mental strain. An interpretation of the results might be that those in the “in work” group may be better equipped by their personality traits to handle stressful situations in their lives. They may be less susceptible to ill health than those on “HL-related sick leave” for both internal and external stressors and have developed coping strategies to handle their life and work situations in relation to HL. As proposed by Raynik et al. [26], it is possible that there is a true association between negative affectivity and mental ill health, at least based on the underlying construct in factor one in the current study. Notably, there were no significant differences between the two groups regarding “hedonic capacity”. However, the variable “negative affectivity” demonstrated an effect size of 0.27, indicating that this variable should probably be considered a stronger risk factor for developing ill health than “hedonic capacity”, being a health factor that alone determines health.

The variable “rest and recovery” also revealed significant differences, suggesting that people in the “in work” state had adequate rest and recovery in their life. There is an established association between the “need for recovery (NFR)” and workers with HL, indicating that this particular target group requires more time to recover after a workday [15, 44]. The reason for this is thought to be a result of increased listening effort and experienced fatigue [45]. In the current project, people on “HL-related sick leave” were younger than those “in work” (*p* <  .05). Consequently, it might be fair to assume that people on sick leave have a busier domestic burden and might not have the possibility to rest and recover to the extent that is required by their HL. Hence, from a health perspective, valuing a person’s total life situation might be important to promote a healthy working life.

### 4.2 Factor 2, Work environment

In Factor 2, work environment, four of the six dimensions of the WEMS [21] clustered: supportive work conditions, internal work experience, autonomy, and reorganization. In addition, management, competence development and satisfaction with salary clustered in this factor. The results demonstrate that those “in work” experienced a more salutary work environment in all measured variables.

The work environment is important for employee well-being in general independent of whether a person has a hearing-related health condition [17]. However, people with HL may experience worse work conditions than employees in general, such as the lack of an accommodating work situation and workplace support [33, 46], which may affect several of the dimensions described above. A literature review by Punch [47] points to a reluctance among employees with HL to request accommodations in the workplace, especially among older employees, employees in part-time work, employees with milder HL and employees who perceive their work environment to be unsupportive. A lack of supportive work conditions may derive from managers’ lack of knowledge about hearing impairment as well as informed managers and coworkers who tend to forget hearing-specific needs in the work situation [46]. To create sustainable work conditions, employees with HL may find that they need to take responsibility for their work situation, such as negotiating hearing-specific accommodations [33], a responsibility that, in itself, can be seen as a contributor to strenuous working conditions [48]. However, if managers are informed about employees’ hearing disabilities, these employees experience good opportunities for adjustments to the work situation [49]. Thus, disclosure of HL may be a promotive factor for work-related health if the workplace is adapted to the hearing-related needs of the employee. Furthermore, the implementation of hearing-specific accommodations could contribute to the career satisfaction and job performance of employees with HL [47], which implies that a supportive work environment with the necessary accommodations influences both productivity and sustainability for employees with HL. This is also evident in this study, where the results demonstrate a positive association between a sustainable work situation (i.e., being in work) and a supportive work environment concerning work conditions, management, and competence development. Based on the cross-sectional study design, it is difficult to determine whether sick leave in this study was caused by an unhealthy work environment or other causes. However, the results are in line with studies of other populations that demonstrate an association between workplace factors and long-term full-time sick leave [50] and the duration of sick leave [51]. Thus, a health-promoting work situation is central to workplace well-being for employees with HL, and a hearing-accommodating work climate (i.e., individual perceptions of a work environment that accommodates hearing-related needs) with supportive work conditions (including management) and opportunities for health-promoting experiences concerning job content (such as autonomy and internal work experience), satisfactory salary, and competence development are salutary factors for employees with HL.

### 4.3 Factor 3, Hearing-related work characteristics and content

This factor includes various work characteristics and content that relates to known difficulties regarding HL. Two of the variables, sound localization and sound discrimination, were first used in a study by Kramer et al. [39], who compared the work performance of persons with and without HL. Kramer et al. found that these two aspects were reported more frequently among persons with HL and concluded that there is a possibility that persons with HL are more aware of these aspects at work. The present study found that individuals ”in work” experienced fewer of these work characteristics compared to those on “HL-related sick leave”. Given that all respondents in the current sample had HL and that the majority used HAs on a regular basis, frequency rather than awareness may be important here. Because of the link between HL and listening effort [52], a possible conclusion might be that listening effort is more difficult in terms of frequency for those on sick leave. Self-perceived listening effort was the major contributor to the phenomenon “need for recovery” investigated by van der Hoek-Snieders et al. [53] and need for recovery was suggested by Nachtegaal [44] to be an underlying factor for sick leave among persons with poor ability to hear in noise. In the current study, persons “in work” were significantly less bothered by noise at work and experienced significantly lower levels of noise at work than those on HL-related sick leave.

### 4.4 Factor 4, Cognitively demanding work content

Five variables loaded on this factor: demanding problem solving, high levels of concentration, conversation with unfamiliar people and/or voices, time pressure (from WEMS) and a high degree of spoken communication. All variables related to work content. One interesting finding is that the amount of oral communication at work did not seem to be related to whether one was “in work” or “on HL-related sick leave”. Modern working life requires flexibility and communication skills, and high pressure is placed on workers to adhere to these norms [54]. In this study, the entire sample was highly educated, and the majority of the respondents in both groups were also involved in jobs that required high education, most commonly in the health and social sector. A reasonable assumption is that they were also involved in job tasks that required a great deal of communication. From current research, it is clear that poorer speech recognition can result in increased listening effort and that persons with HL consequently expend a large amount of listening effort in communication [e.g., 55]. However, in the current study, not all variables in this factor related to listening and communication. Research findings show that one important explanation for experienced fatigue is the increased concentration effort that is required to solve different tasks [discussed in 56]. In the current study, the work content of those “in work” involved significantly lower levels of concentration, and they also experienced significantly less fatigue (see discussion, Factor five). Hence, the level of concentration one must mobilize at work might be related to the position in the labor market (i.e., “in work” or on “HL-related sick leave”) rather than the amount of oral communication involved.

### 4.5 Factor 5, Hearing loss-related symptoms

Tinnitus, sound fatigue, headache (and migraine), fatigue and dizziness loaded on this group, and significant differences were found for all these variables between the two groups. Most of these health variables are known to be comorbid with HL. Previous studies have concluded that fatigue is related to HL, probably as a result of the increased cognitive load in relation to listening effort [e.g., 57]. However, as Holman et al. [58] established in their systematic review of the relationship between HL and fatigue, the area is still in its infancy because of the few studies published and the heterogeneity regarding outcome measures of fatigue. In the current study, fatigue was assessed with only one item; consequently, a deeper analysis of this phenomenon is not possible. However, given that there were no differences between the two groups regarding exposure to spoken communication (see discussion, Factor four), listening effort alone might not explain the increased levels of fatigue that individuals on “HL-related sick leave” experience.

In recent years, a hearing health aspect, “sound-induced auditory fatigue”, has been identified in research on noise exposure. Studies have found a connection between communication-intense jobs, such as midwives and preschool teachers, and sound-induced auditory fatigue [40, 59]. Not only do these kinds of jobs involve high levels of noise, but the noise itself consists of high levels of information that workers must pay attention to. Informants discussed this issue in a qualitative study where employees in communication-intense workplaces such as health care and preschools were asked how they experienced their sound environment. The authors found that the informants considered workplace noise meaningful but disturbing [60]. Preschool teachers explained that they “needed to be constantly attentive to sounds, in order to be aware of what was going on among the children” [60, p.6]. In the current study, people on “HL-related sick leave” claimed that they were bothered by noise at work and experienced auditory sound fatigue to a higher extent than those “in work”. However, this matter cannot be tied to the type of job (Table 2). Rather, this systematic pattern might be a result of the working conditions and the work content in a specific workplace.

### 4.6 Factor 6, Energy-demanding activities

Three variables loaded on this factor: nonverbal communication strategies, verbal communication strategies and energy-demanding tasks outside of work. The first two variables are indices from the CSS [41]. Interestingly, persons “in work” used fewer verbal and nonverbal communication strategies in their daily life compared to those on “sick leave”. The CSS is not designed for working life, and the respondents were thus asked to view the usage of communication strategies in their daily life (not just in working life). Information/education about communication strategies are among the main elements in audiological rehabilitation (AR) [61], and these strategies are viewed as core features of coping mechanisms for persons with HL. However, anecdotal reports on how communication strategies are taught to patients in clinical practice suggest that the focus is educating patients on *how* to use them rather than *when* to use them. In a qualitative study on HL and daily life fatigue by Holman et al. [62], the authors found that the coping strategies that the informants talked about (in relation to difficult listening situations) focused on reducing efforts by withdrawal, avoidance or planning. The informants stated, for example, that topics must be worth listening to or they would withdraw from the situation. This coping behavior might be similar for the population in the current study. One suggestion for the finding in this study is that persons “in work” spend less energy and effort on activities in their daily life compared to those on “HL-related sick leave”, consciously or unconsciously, by using fewer verbal and nonverbal communication strategies.

### 4.7 Factor 7: Bodily pain and ache

The last factor contained three variables, but only one demonstrated significant differences between the two groups, “pain in shoulders and neck”. From clinical experience and anecdotal information, many adults with HL in working life suffer from pain in the shoulders and neck, probably due to tension in the muscles. Many adults with HL put considerable effort into trying to comprehend speech, especially in noisy situations. Interestingly, bodily pain in adults with HL has received limited research attention. Physical health problems that have been identified in previous research have included pain and tension in the neck [8, 11, 12, 63], aspects that were also highlighted in the current study. In previous studies, women with HL in working life have been identified as a vulnerable group with regard to pain. Coniavitis Gellerstedt et al. [64] found that compared to men, women more frequently had problems with the neck and back and headaches. Gender aspects in relation to pain are beyond the scope of the current study; however, these matters need to be addressed in future studies.

## 5 Conclusions

The current study investigated health factors for a sustainable work situation in relation to HL by comparing two groups: workers with HL “in work” and those on “HL-related sick leave”. Comparative analyses revealed significant differences between the two groups in the majority of the investigated variables. Individuals “in work” experienced less mental strain, a healthier work environment, less hearing-related work characteristics and content, less cognitively demanding work content, fewer HL-related symptoms, less energy-demanding activities, and less bodily pain compared to those on “HL-related sick leave”. The results demonstrate a clear pattern regarding health factors for a sustainable working life. The type of job was not related to whether a respondent was on sick leave or in work. Rather, the working climate and the content of the work mattered. Given that this study embraces a deductive approach to salutary factors in working life for the target group, conducting additional inductive qualitative interviews to explore aspects that promote a healthy working life in relation to HL would deepen our knowledge regarding sustainable work situations.

### 5.1 Strengths and limitations

A strength of this study is its health focus (i.e., the salutogenic perspective). As previously highlighted, many studies in the field (HL in working life) often focus on issues related to ill-health and problems experienced in the working life. However, as health and ill-health are non dichotomous concepts, a successful approach in clinical practice may involve learning from individuals who function well in the working life. Consequently, the results of this study provide valuable guidance to clinicians and other stakeholders on how to design hearing health care interventions to prevent ill-health and reduce sick leave among workers with HL.

Another strength in this study is its sample size. The results are based on response from approximately 495 participants, which is higher compared to other studies in the field with similar design (HL in working life). Furthermore, the respondents are part of a clinical population. This approach is somewhat rare within the field, where many cross-sectional surveys are based on individuals with self-assessed HL. Notably, the effect sizes in the analyzes were small, and the results must therefore be interpreted with caution. In addition, the two groups differed in sample size, and the HL-related sick leave group was quite small. Despite these limitations, the results reveal a consistent and systematic pattern in which the “in work” group was better off in regard to health-related factors, which might support the theoretical foundations of this study.

## Ethical approval

The current study was approved by the Swedish Ethical Review Authority, Dnr 2017/150.

## Informed consent

All participants in the current study have given written consent to study participation.

## Conflict of interests

The authors report no conflicts of interests.
